# Sleep disorders among university students as underestimated mental health problem

**DOI:** 10.1192/j.eurpsy.2024.1609

**Published:** 2024-08-27

**Authors:** E. L. Nikolaev, T. Nikolaeva, M. Alhasan

**Affiliations:** ^1^Department of Social and Clinical Psychology; ^2^Medical Faculty, Ulianov Chuvash State University, Cheboksary, Russian Federation

## Abstract

**Introduction:**

The effect of sleep disorders on the weakening of the students’ mental health potential is still underestimated. Students might not openly complain of having problems with sleep, considering them insignificant. Nevertheless, sleep disorders may be the sign of actual or developing mental health problems.

**Objectives:**

To reveal the prevalence of parasomnic and insomnic disorders in university students, who do not have health related complains.

**Methods:**

We surveyed 77 first and second-year students of both genders by means of a questionnaire that included questions describing the signs of various sleep disorders.

**Results:**

One third of the students revealed having parasomnic disorders in the form of dissociated sleep states – 35.1% of the respondents talk in sleep (states of somniloquy or sleep talking), 6.5% get seated on their beds, 5.2% get up from their beds (states of partial awakenings and confusional arousals), 5.2% walk around the room or house (sleepwalking, or somnambulism). Over half of the students experience night phobias (53.2%), 2.6% out of them experience them constantly. Some students’ fears grow into nightmares. Half of the respondents (50.6%) state they very rarely see nightmares. Every fifth student (20.8%) sees nightmares only from time to time. 10.4% of the students see them very often or constantly.Over half of the respondents (55.8%) complain of insomnic disorders in the form of insomnia. 3.9% of them experience it constantly, 10.4% – often, 16.9% – sometimes, and 24.7% – rarely

**Conclusions:**

The frequency of sleep disorders in students is very high. Consequently, it is important to inform university students timely about potential risks and ways to avoid them.

**Disclosure of Interest:**

None Declared

